# Supplementary material for: Evaluation of a nursing and midwifery exchange between rural and metropolitan hospitals: A mixed methods study
Source: PLoS One. 2020 Jul 1;15(7):e0234184. doi: 10.1371/journal.pone.0234184 (PMC7329084; doi:10.1371/journal.pone.0234184)
Supplement: S1 File — (PDF) [file pone.0234184.s001.pdf]

Consolidated criteria for reporting qualitative studies (COREQ): 32-item checklist

Domain 1: Research team and reflexivity	Response (and manuscript line and page number)
Personal Characteristics	
1. Interviewer/facilitator. Which author/s conducted the interview or focus group?	Page 6
2. Credentials. What were the researcher's credentials? E.g. PhD, MD	These have been included in the submission information
3. Occupation. What was their occupation at the time of the study?	These have been included in the submission information
4. Gender. Was the researcher male or female?	These have been included in the submission information
5. Experience and training. What experience or training did the researcher have?	These have been included in the submission information
Relationship with participants	
6. Relationship established. Was a relationship established prior to study commencement?	Page 6
7. Participant knowledge of the interviewer<br>What did the participants know about the researcher? e.g. personal goals, reasons for doing the research	Page 6
8. Interviewer characteristics. What characteristics were reported about the interviewer/facilitator? e.g. Bias, assumptions, reasons and interests in the research topic	Page 6

Domain 2: study design	
Theoretical framework	
9. Methodological orientation and Theory. What methodological orientation was stated to underpin the study? e.g. grounded theory, discourse analysis, ethnography, phenomenology, content analysis	Page 11- Thematic Analysis
Participant selection	
10. Sampling. How were participants selected? e.g. purposive, convenience, consecutive, snowball	Page 5- all NN within participating health services were asked to consent to participate.
11. Method of approach. How were participants approached? e.g. face-to-face, telephone, mail, email	Page 6
12. Sample size. How many participants were in the study?	Page 6
13. Non-participation. How many people refused to participate or dropped out? Reasons?	N/A
Setting	
14. Setting of data collection. Where was the data collected? e.g. home, clinic, workplace	Page 6-7
15. Presence of non-participants. Was anyone else present besides the participants and researchers?	8

16. Description of sample. What are the important characteristics of the sample? e.g. demographic data, date	Page 8
Data collection	
17. Interview guide. Were questions, prompts, guides provided by the authors? Was it pilot tested?	Page 7-8
18. Repeat interviews. Were repeat interviews carried out? If yes, how many?	N/A
19. Audio/visual recording. Did the research use audio or visual recording to collect the data?	N/A
20. Field notes. Were field notes made during and/or after the interview or focus group?	N/A
21. Duration. What was the duration of the interviews or focus group?	N/A
22. Data saturation. Was data saturation discussed?	N/A
23. Transcripts returned. Were transcripts returned to participants for comment and/or correction?	N/A

Domain 3: analysis and findings	
Data analysis	
24. Number of data coders.<br>How many data coders coded the data?	Page 11
25. Description of the coding tree. Did authors provide a description of the coding tree?	N/A
26. Derivation of themes. Were themes identified in advance or derived from the data?	Page 11-12
27. Software. What software, if applicable, was used to manage the data?	N/A
28. Participant checking. Did participants provide feedback on the findings?	N/A
Reporting	
29. Quotations presented. Were participant quotations presented to illustrate the themes / findings? Was each quotation identified? e.g. participant number	Page 12-13
30. Data and findings consistent. Was there consistency between the data presented and the findings?	Page 12-13
31. Clarity of major themes. Were major themes clearly presented in the findings?	Page 13
32. Clarity of minor themes. Is there a description of diverse cases or discussion of minor themes?	N/A
