# Supplementary material for: Evaluation of a nursing and midwifery exchange between rural and metropolitan hospitals: A mixed methods study
Source: PLoS One. 2020 Jul 1;15(7):e0234184. doi: 10.1371/journal.pone.0234184 (PMC7329084; doi:10.1371/journal.pone.0234184)
Supplement: S2 File — (PDF) [file pone.0234184.s002.pdf]

Table: checklist of the Conducting and Reporting of Delphi Studies (CREDES)

		Page location in the main document
**Rationale for the choice of the Delphi technique**	Justification	7
**Planning and design**	Planning and process	7
	Definition of consensus	15
**Study conduct**	Information input	15
	Prevention of bias	20
	Interpretation and processing of results	16-22
	External validation	16-22
**Reporting**	Purpose and rationale	14
	Expert panel	7
	Description of the methods	14
	Procedure	15-16
	Definition and attainment of consensus	15
	Results	16-22
	Discussion of limitations	27
	Adequacy of conclusions	20-22
	Publication and dissemination	20-22
