# Supplementary material for: Evaluation of a nursing and midwifery exchange between rural and metropolitan hospitals: A mixed methods study
Source: PLoS One. 2020 Jul 1;15(7):e0234184. doi: 10.1371/journal.pone.0234184 (PMC7329084; doi:10.1371/journal.pone.0234184)
Supplement: Attachment — Submitted filename: Response to Feedback.docx [file pone.0234184.s003.docx]

To the Editor	29/03/2020

Thank you for considering this second submission of *Evaluation of a nursing and midwifery exchange between rural and metropolitan hospitals: a mixed methods study.* We thank you for the feedback and have made the changes as suggested below.

| **Feedback** | **Amendment** |
| --- | --- |
| Consider the journal's publication criteria, specifically in reporting the methods of the qualitative component of your study. It's recommended authors use a relevant checklist listed by the Equator Network such as the SRQR to ensure complete reporting of qualitative studies, including your qualitative methodology and theory, and further detail on your methods of analyses. | Thank you for your feedback. We have attached a COREQ checklist for the thematic analysis of data and made changes to reflect the requirements of the checklist  For the Delphi- we have used the CREDES checklist |
| As this article is not a systematic/or literature review article, it's inappropriate to report some of the results of your integrative review, as this is being published elsewhere. Instead you could include a summary of the literature in the background of the article | The summary of the literature review has been removed from the manuscript. Phase two now references the review undertaken only. |
| Include the response rates for surveys | This data is not available however all other data is included (numbers in each cohort, level of practice etc) |
| Your justification for your quantitative analysis methods is in the limitations section, this would be better placed earlier in the methods section | This has been moved to the appropriate section |

We appreciate the opportunity to resubmit.

Many thanks

Amy-Louise Byrne (Corresponding author)
