# Supplementary material for: Evaluation of a nursing and midwifery exchange between rural and metropolitan hospitals: A mixed methods study
Source: PLoS One. 2020 Jul 1;15(7):e0234184. doi: 10.1371/journal.pone.0234184 (PMC7329084; doi:10.1371/journal.pone.0234184)
Supplement: Attachment — Submitted filename: Reponse to Reviewers.docx [file pone.0234184.s004.docx]

Plos One	20^th^ May 2020

To the Editor

Thank you for the opportunity to respond to reviewers re manuscript: *Evaluation of a nursing and midwifery exchange between rural and metropolitan hospitals: a mixed methods study.*

We have provided detailed feedback of the changes made in the table below. We appreciate the time and efforts for the reviewers and editors.

| **Feedback** | **Changes** |
| --- | --- |
| Please ensure that your manuscript meets PLOS ONE's style requirements, including those for file naming | Amended as per style guide  Supporting information file names changed |
| Please amend the manuscript submission data (via Edit Submission) to include author Elspeth Wood. |  |
| Please ensure that you refer to Figure 1 in your text as, if accepted, production will need this reference to link the reader to the figure. | There is now a reference to figure 1 in the body. ‘Figure 1 below shows participants likelihood of leaving their job in nursing’ |
| Please include captions for your Supporting Information files at the end of your manuscript, and update any in-text citations to match accordingly. Please see our Supporting Information guidelines for more information [https://protect-au.mimecast.com/s/BwAsCxnMZWUDXKYycR5-NP?domain=journals.plos.org](about:blank) | Reference to Appendix removed as all tables are embedded in body of document.  Supporting information is now referred to at the end of the manuscript as per the style guide.  S1 Fig- COREQ Checklist  S2 Fig- CREDES Checklist |
| Abstract should be modified: increase the presentation of numerical results and cut-short the discussion or mention the conclusion only. | Cohort numbers included in methods  Statistical analysis summary included in Results |
| Under the introduction, the state were the study was carried out should be stated. The authors should clarify whether the last portion of the abstract is the conclusion or discussion. | Queensland, Australia added.  Heading changed to conclusion |
| This should be fused with the background as a large portion of the contents of the background(page 5 lines 98-121) is repeated in the introduction. | Background information on NMEP has been reduced and some geographical changes made to better flow the ideas of the background |
| PAGE 3,LINE 67 QECD SHOULD 1ST BE WRITTEN IN FULL. | This is now amended and reads; ‘Organisation for Economic Cooperation (OECD)’ |

Many thanks

Amy-Louise Byrne (Corresponding author).
